# The Efficacy and Safety of a Human Perirenal Adipose Tissue-Derived Stromal Vascular Fraction in an Interstitial Cystitis Rat Model

**DOI:** 10.1007/s13770-022-00505-w

**Published:** 2023-01-04

**Authors:** Ji Yong Ha, Eun Hye Lee, So Young Chun, Jun Nyung Lee, Yun-Sok Ha, Jae-Wook Chung, Bo Hyun Yoon, Minji Jeon, Hyun Tae Kim, Tae Gyun Kwon, Eun Sang Yoo, Bum Soo Kim

**Affiliations:** 1 Department of Urology, Dongsan Medical Center, Keimyung University School of Medicine, Daegu, Republic of Korea; 2 Joint Institute for Regenerative Medicine, Kyungpook National University, Daegu, Republic of Korea; 3 BioMedical Research Institute, Kyungpook National University Hospital, Daegu, Republic of Korea; 4 Department of Urology, School of Medicine, Kyungpook National University, Kyungpook National University Hospital, Daegu, 41944 Republic of Korea; 5 Department of Urology, School of Medicine, Kyungpook National University, Chilgok Kyungpook National University Hospital, Daegu, Republic of Korea

**Keywords:** Interstitial cystitis, Animal model, Adipose tissue, Stem cell, Stromal vascular fraction

## Abstract

**Background:**

Interstitial cystitis (IC) is a chronic and intractable disease that can severely deteriorate patients’ quality of life. Recently, stem cell therapy has been introduced as a promising alternative treatment for IC in animal models. We aimed to verify the efficacy and safety of the human perirenal adipose tissue-derived stromal vascular fraction (SVF) in an IC rat model.

**Methods:**

From eight-week-old female rats, an IC rat model was established by subcutaneous injection of 200 μg of uroplakin3A. The SVF was injected into the bladder submucosal layer of IC rats, and pain scale analysis, awakening cytometry, and histological and gene analyses of the bladder were performed. For the* in vivo* safety analysis, genomic DNA purification and histological analysis were also performed to check tumorigenicity and thrombus formation.

**Results:**

The mean pain scores in the SVF 20 μl group were significantly lower on days 7 and 14 than those in the control group, and bladder intercontraction intervals were significantly improved in the SVF groups in a dose-dependent manner. Regeneration of the bladder epithelium, basement membrane, and lamina propria was observed in the SVF group. In the SVF groups, however, bladder fibrosis and the expression of inflammatory markers were not significantly improved compared to those in the control group.

**Conclusion:**

This study demonstrated that a perirenal adipose tissue-derived SVF is a promising alternative for the management of IC in terms of improving bladder pain and overactivity.

**Supplementary Information:**

The online version contains supplementary material available at 10.1007/s13770-022-00505-w.

## Introduction

Interstitial cystitis (IC), an idiopathic chronic inflammatory condition of the urinary bladder, is an intractable disease that causes serious deterioration of patients’ quality of life [1]. In South Korea, the prevalence of IC was estimated to be 0.2–0.3%, with females being more affected [2]. Various treatment modalities, such as oral drugs, intravesical hyaluronic acid instillation, and bladder hydrodistention, are currently available, but no treatments guaranteeing a definite cure for IC are currently available [3]. To overcome these limitations, stem cell therapy is considered a promising alternative treatment for IC. Since 2010, a number of researchers have used preclinical studies to analyze the efficacy of stem cells in treating IC [4–7].

Although the efficacy of mesenchymal stem cells (MSCs) from various cell sources, such as adipose tissue, bone marrow, urine, amniotic fluid, and umbilical cord blood, for IC has been verified in previous studies, they have not yet been applied in clinical settings due to a lack of comprehensive understanding of cell reactions in the human body. In addition, some MSCs, such as urine, amniotic fluid, and umbilical cord blood-derived stem cells, have limitations for application in humans in terms of efficacy and safety. The complicated process of cell preparation, including the need for cell separation and culture conditions, is also a hurdle for the early clinical application of MSCs.

As adipose tissue-derived stem cells (ADSCs) have shown promise in regenerative medicine, they have gained popularity as a major cell source since they were first characterized in 2001 [8, 9]. With the advancement of ADSC-related research, simpler and more effective products are needed for easy clinical application. The stromal vascular fraction (SVF), which includes ADSCs, endothelial precursor cells, endothelial cells, macrophages, smooth muscle cells, lymphocytes, and pericytes, has gained attention as an alternative to ADSCs [9]. The advantages of SVF over ADSCs are that it has not only the characteristics of ADSCs but also distinctive and heterogeneous cellular components, which may induce better therapeutic effects. Moreover, easier preparation, including instantaneous acquisition and minimal contact with reagents, is also one of the advantages of the SVF [9].

Currently, an SVF is usually procured and isolated from subcutaneous adipose tissue via liposuction [10]. More recently, perirenal adipose tissue has been proposed as a potential cell source for regenerative medicine [11]. Perirenal adipose tissue contains various cells, such as stem cells, adipocytes, fibroblasts, vascular cells, neural cells, inflammatory cells, and immune cells [12]. Perirenal adipose tissue includes many brown adipocytes and also shows high conversion efficiency of beige cells from white adipocytes [11]. However, most perirenal adipose tissues are discarded after numerous kidney surgeries. In addition, autologous perirenal adipose tissue is not always available in all patients because it can only be procured through retroperitoneal perirenal dissection, which is more invasive than liposuction. Owing to these characteristics of perirenal adipose tissue, a perirenal adipose tissue-derived SVF is considered to be more useful if the allogenic origin can be used. In this study, we aimed to isolate an SVF from human perirenal adipose tissue and verify the efficacy and safety of the SVF in a rat model of IC.

## Materials and methods

### Preparation of the SVF

Donated perirenal fat was collected at random from 92 donors and used in the experiments. Before use, acquired perirenal fat tissues were delivered to the laboratory in ice cold conditions and kept at − 80 °C. We purchased a Ustem kit (Ustem Biomedical, Seoul, Korea) to obtain the final SVF. The Ustem kit is a disposable medical device used for adipose stem cell isolation (SVF extraction). Twenty-five grams of human perirenal adipose tissues were used as an initial amount and the final SVF was obtained according to the manufacturer’s protocol. Equal volumes of 0.1% of collagenase I solution and chopped perirenal fat were added to the upper case and incubated at 37 °C for 25 min while shaking at 200 RPM. After incubation, the kit was centrifuged at 1,600 RPM for 8 min. The separated fat and debris in the upper case were discarded, and the SVF was acquired in the lower case. The upper case was filled with 40 ml of normal saline and centrifuged at 1,500 RPM for 5 min to wash the extracted SVF. This washing step was repeated three times. The washed SVF was collected in a 21G needle syringe and filtered with a Ustem micro filter. The final volume was 1 ml.

### Generation of the IC animal model

IC in a painful bladder animal model was generated via injection of 200 μg of uroplakin3A (MyBiosource, San Diego, CA, USA) to eight-week-old female Sprague–Dawley rats subcutaneously after a week of animal stabilization [13]. To optimize the dose of the SVF, all rats were divided into four groups (Sham group, IC + 5 ul SVF group, IC + 10ul SVF group, IC + 20ul SVF group, n = 5) according to the injection dose of the SVF to the bladder submucosa: phosphate-buffered saline (PBS) injection (control) and 5, 10, and 20 μl of the SVF.

**Fig. 1:**
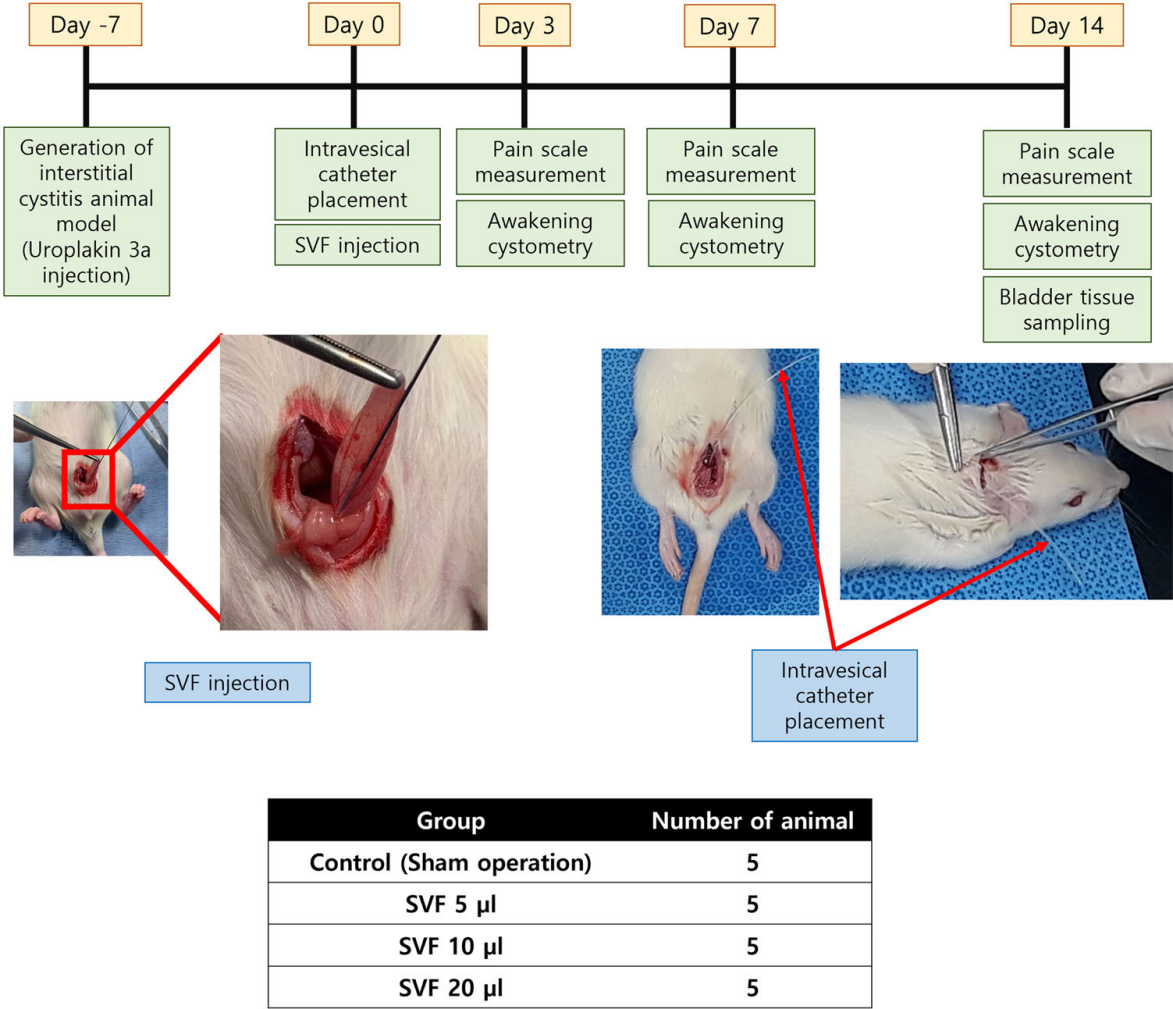
*In vivo* study protocol for the optimization of the dose of the SVF **A**. Experimental flow for the generation of IC animal model, SVF injection and outcome analysis **B**. Animal grouping for the comparison of efficacy of each SVF dose group. SVF; stromal vascular fraction, IC; interstitial cystitis

One week after the generation of the IC rat model, all rats were anesthetized via injection of 0.1 mg/kg of zoletil into the right gluteus muscle. The bladder was exposed with a 2-cm lower midline abdominal incision, and the bladder dome was punctured. The proximal end of a polyethylene-50 (PE) tube (Natsume Seisakusho Co., Tokyo, Japan) was introduced into the bladder, and the puncture site of the bladder was tightened. The distal end of the PE tube was inserted into the subcutaneous tunnel of the left dorsum and was extracted around the posterior neck. After confirming that the urine was well drained through the PE tube, the tube was fixed at both the bladder and posterior neck sites, and the abdominal wound was sutured. According to each group, 10 μl of PBS (control) and 5, 10, or 20 μl of the SVF were injected into the four sites of the bladder submucosa, including the bladder anterior wall, dome, and both lateral walls, using a Hamilton syringe on the same day as PE tube insertion before abdominal wound suturing. The* in vivo* study protocol is shown in Fig. 1.

### Optimization of the injection dose of the SVF for the treatment of IC

#### Pain scale measurements

The rat grimace scale (RGS) was used to quantify the pain level of IC rats [14]. The action units of the RGS include whisker changes, ear changes, nose/cheek flattening, and orbital tightening, and the pain score of each action unit consists of 0 (not present), 1 (moderate), and 2 (obvious). Each rat was placed in a metabolic cage one by one on days 3, 7, and 14 after the injection of PBS or the SVF. A digital video camera was placed in front of the cage to record the facial expressions of the rats, which were digitally recorded for 30 min. Video files were captured and used to focus on the visibility of the rats’ faces. From each three-minute interval, the single shot that was the most feasible for RGS evaluation was manually selected by one investigator, and all the images were evaluated by three investigators. SVF injection was performed a week after uroplakin 3A injection. The final score was determined by the consensus of three investigators. The mean values of the total pain scores of each action unit were compared.

#### Awakening cystometry

Three of five rats from each group, in which the PE tube was well-maintained without obstruction or breakage, underwent awakening cystometry on days 3, 7, and 14 after injection of PBS or the SVF. The rats were placed in a metabolic cage without anesthesia, and the distal end of the PE tube was connected to a pressure transducer. Sterile saline was infused at a constant rate of 40 μl/min using a syringe pump system. Intravesical pressure and bladder intercontraction intervals were measured using a Power Lab System (AD Instruments Pty., Ltd., Bella Vista, NSW, Australia).

#### Histological analyses

On day 14, after injection of PBS or the SVF, immediately after performing awakening cystometry, all rats were euthanized and the bladders were procured. Half of each bladder was fixed in 4% paraformaldehyde, and the paraffin-embedded samples were cut into 5-μm sections for hematoxylin and eosin (H&E), Masson’s trichrome, and toluidine blue staining for histological, fibrotic, and mast cell infiltration analysis, respectively. Bladder mucosal damage or regeneration and submucosal cellular infiltration were evaluated using H&E staining. The bladder sections were then washed with xylene to remove the paraffin, and the slides were treated with an ethanol series for toluidine blue staining. Slides were stained with toluidine blue for 4 min, treated with an ethanol series, and mounted for mast cell counting. Finally, the deparaffinized and rehydrated sections were re-fixed in Bouin’s solution, stained with Weigert’s iron hematoxylin working solution and Biebrich scarlet-acid fuchsine solution, and then differentiated in phosphomolybdic–phosphotungstic acid solution until the collagen turned red for Masson’s trichrome staining. The slides were transferred to an aniline blue solution and differentiated in a 1% acetic acid solution. Collagen fibers stained blue.

Immunohistochemical (IHC) analysis was performed using CD3, CD31, UPK3, and ZO-1 antibodies (Abcam, Cambridge, UK; 1:200 dilution) to observe the cytotoxic T-cells, endothelial cells, inner membrane, and intracellular side of the plasma membrane. The deparaffinized sections were treated with 0.2% Triton-X for 10 min before being incubated in 200 μl of 5% PBS for 2 h. The sections were incubated with the primary antibody overnight at 4 °C and then with the secondary antibody (Alexa Fluor 594 goat anti-rabbit IgG, Abcam, 1:1000 dilution) for 1 h at room temperature.

#### Gene expression analysis

The remaining half of each bladder was used for real-time polymerase chain reaction (PCR) analysis. The entire ribonucleic acid (RNA) was separated using the RNeasy Mini Kit (QIAGEN, Valencia, CA, USA), and complementary deoxyribonucleic acid (cDNA) was prepared using reverse-transcription Reagents (Applied Biosystems, Carlsbad, CA, USA) according to the manufacturer’s instructions. Gene expression analysis was performed using a real-time PCR device and 7300 System SDS Software (Applied Biosystems). The PCR conditions for using SYBR® Green PCR Master Mix (Bio-Rad, Hercules, CA, USA) were 95 °C for 10 min, followed by 45 cycles of 95 °C for 10 s, 58 °C for 50 s, and 72 °C for 20 s. To analyze relative changes in gene expression, the Ct value for the target gene was normalized to that of its endogenous control and transformed to a relative gene expression value using the 2-^ΔΔ^Ct method.

### *In vivo* safety of the SVF

#### Generation of animals

The Balb/c nude mice (male, six-week-old, Joongang Experimental Animal, Seoul, Korea) were provided with water and food ad libitum. A total of 36 nude mice were used in the experiment (Sham group = 1, IC group = 3, IC + SVF-injected group = 5 on each scarifying day), and animals were sacrificed on days 3, 7, 14, and 28 after SVF injection. All animals were kept in a controlled specific pathogen-free environment with a 12-h light and dark cycle, temperature of 25.0 ± 0.2 °C, and a humidity of 45 ± 2%. Uroplakin3A was purchased from MyBioSource (MBS1409416, San Diego, CA, USA) and injected subcutaneously at a dose of 200 μg. SVF was injected at four bladder sites (anterior, posterior, right, and left). All animals were sacrificed with CO_2_ gas, according to the Animal Care and Use Committee guidelines, at the end of the experiment.

#### Genomic DNA purification

The obtained organs (bladder, muscle, liver, heart, lung, spleen, kidney, blood, brain, ear, tail, ovary, tooth, and bone marrow) were washed with 1 × PBS prior to genomic DNA extraction. Genomic DNA was extracted using an automated system (Maxwell® RSC, Promega, Madison, WI, USA) and a DNA extraction kit (Maxwell® RSC tissue DNA kit). These steps were performed according to the manufacturer’s instructions. Real-time PCR was performed using an ABI Step One Plus detection system (Applied Biosystems, Foster City, CA, USA) with SYBR Green (Luna Universal qPCR Master Mix, New England BioLabs Inc., Ipswich, MA, USA). The primer sequences used for the human Alu primer were as follows: forward 5′ CTG GGC GAC AGA ACG AGA TTC TAT 3′; reverse 5′ CTC ACT ACT TGG TGA CAG GTT CA 3′. qPCR was performed with 38 cycles of denaturation at 95 °C for 5 s and annealing at 60 °C for 20 s.

#### Histological analyses

Sampled organs (bladder, liver, heart, lung, spleen, and kidney) were fixed in 10% formalin prior to histological analysis. Paraffin-embedded samples were cut into 5-μm-thick sections. H&E staining was performed to determine the epithelial structure. For H&E staining, deparaffinized tissue slides were stained with hematoxylin for 10 min and with 0.5% eosin for 3 min. Toluidine blue staining and Masson’s trichrome staining were performed to visualize mast cells and collagen, respectively. Mast cells were stained by putting toluidine blue solution on deparaffinized tissue slides for 3 min and then rapidly washing them in 95% alcohol. To visualize collagen, deparaffinized tissue sections were stained in Weigert’s hematoxylin for 15 min, in Biebrich scarlet-acid fuschin for 15 min, in 2.5% phosphomolybdic–phosphotungstic acid solution for 15 min, and in 2.5% aniline blue solution for 12 min in this order. The HuNu, CD3, CD31, UPK3, and ZO-1 antibodies were purchased from Abcam (Cambridge, MA, USA). Incubation with each primary antibody was performed for 24 h at 4 °C, and a secondary antibody (Alexa Fluor 594; Life Technology, Waltham, MA, USA) was applied for 1 h at room temperature. 4′,6-diamidino-2-phenylindole (DAPI) was used to stain the nuclei. The primer sequences used are listed in Table 1.

**Table 1:** Primer sequences

Gene	Sequences	
	Forward	Reverse
*GAPDH*	5′ CAA GTT CAA CGG CAC AGT CA 3′	5′ CCC CAT TTG ATG TTA GCG GG 3′
*UP1a*	5′ TAG ATG CAC CTT GGC CTG AA 3′	5′ GGA AAA TGG AGG GAG GTG GA 3′
*UP1b*	5′ TCC GTC AGA CTG GCA GAA AT 3′	5′ GTC CAG GTT GAG AGG CTC TT 3′
*UP2*	5′ AGC CTG TTA ATT GCC TTG CC 3′	5′ AGG CAC CAC AAA GTC TGA CT 3′
*CK7*	5′ TTC CCC GAA TCT TTG AGG CT 3′	5′ TCT TCC ACC ACA TCC TGC AT 3′
*DK13*	5′ GTC AAT GTG GAG ATG GAC GC 3′	5′ CTC TGC ACT CTT GGT CTG GA 3′
*CK18*	5′ TGC AGA AGA CAA CTA CCC GT 3′	5′ ATT GGT CCC TCA GTT CCC AG 3′
*CK19*	5′ TGT CGA CCT AGC CAA GAT CC 3′	5′ TCA GCA TCC TTC CGG TTC TT 3′
*Pan-CK*	5′ TGA ATG GCC ACT GAA GTC CT 3′	5′ CTC GGA CGG GTC TCT AGT TC 3′
*Pax7*	5′ ACCAAGCTTTCAAGTCCGCA 3′	5′ GCCTTACATTCAGGAGGATG 3′
*MyoD*	5′ TTC CGG AGT GGC AGA AAG TTA A 3′	5′ TCA AGT CTA TGT CCC GGA GTC G 3′
*Desmin*	5′ ACGTTATTTTGCCTGGATCG 3′	5′ GGGATGGGGACAGTCCTATT 3′
*Myosin*	5′ ACA AGG AGC AGG CAG AGA AA 3′	5′ TCA CRG GCT TTG GTT CCA TT 3′
*a-SM actin*	5′ TGC TTC CTC CTC CTC CTT TG 3′	5′ TCC TTG GAA GTA CTG CCG TT 3′
*CD31*	5′ CTG CCA GTC CGA AAA TGG AAC 3′	5′ CTT CAT CCA CCG GGG CTA TC 3′
*CD34*	5′ GTCACCTCTGGAGTTCTGCT 3′	5′ CACCCAGCCTTTCTCCTGTA 3′
*CD73*	5′ TCC TGG GCT ACG ATG CTA TG 3′	5′ ATC AGT CCT TCC ACA CCG TT 3′
*CD105*	5′ GCC CAC TTC TCC TGA CTT CT 3′	5′ TAT CCA GAG GTA AGG CTG CG 3′

### Statistical analysis

All data are presented as the mean. Differences in pain scores, bladder intercontraction intervals, number of mast cells, and quantitative PCR outcomes were analyzed using Student’s t-test and one-way analysis of variance (ANOVA). When the value was revealed to be significant by ANOVA, Tukey’s post-hoc test was performed. Statistical results were calculated using IBM SPSS Statistics for Windows, version XX (IBM Corp., Armonk, N.Y., USA), and statistical significance was set at p < 0.05.

### Results

#### Optimal dose of the SVF for IC rats

##### Pain scale

**Fig. 2:**
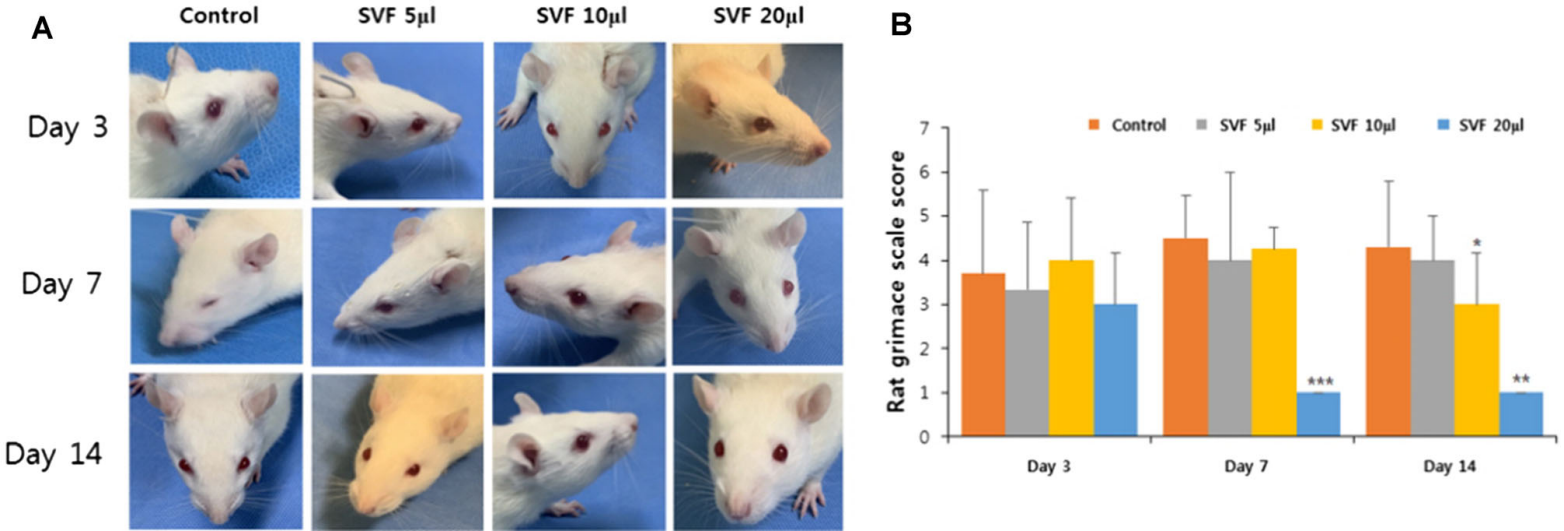
Comparison of pain scores evaluated by the rat grimace gcale (RGS). **A**, **B** The mean RGS pain scores of the SVF 20 μl group at day 7 and 14 were significantly lower than those of the control group. ** and *** above the bars indicate significant differences at *p* < 0.01. * indicates significant differences at *p* < 0.05

To verify the efficacy of the SVF for bladder pain and to optimize the appropriate SVF dose, the pain scores of IC rats were evaluated using the RGS on days 3, 7, and 14 after SVF injection. On day 3, the mean pain scores of all SVF groups were not significantly improved compared to those of the control group (control, 3.50; SVF 5 μl, 3.80; SVF 10 μl, 1.71; SVF 20 μl, 2.17, p = 0.102). However, the mean pain score of the SVF 20 μl group was significantly improved at days 7 (0.60) and 14 (0.60) compared to that of the control group (4.42 and 4.33, p = 0.004). While the SVF 5 μl group did not show a statistical difference in mean pain scores in the entire study period compared to those of the control group, the SVF 10 μl group showed a statistical improvement in mean pain scores at day 14 compared to those of the control group (3.00, 2.43, p = 0.025) (Fig. 2).

##### Awakening cystometry

To analyze the functional improvement of the SVF, awakening cystometry was performed, including in the control group. The intercontraction intervals of the bladder were measured on days 3, 7, and 14 after the SVF or PBS injection. On day 3, all SVF injection groups showed significantly longer mean bladder intercontraction intervals than the control group, and these differences were further increased in a dose-dependent manner (control, 51.2; SVF 5 μl, 112.0; SVF 10 μl, 158.2; SVF 20 μl, 184.0, p = 0.011). These findings were similarly observed at day 7 (control, 72.0; SVF 5 μl, 137.4; SVF 10 μl, 175.0; SVF 20 μl, 266.4, *p* = 0.008) and 1c (control, 60.2; SVF 5 μl, 144.8; SVF 10 μl, 185.0; SVF 20 μl, 296.8, *p* = 0.007) (Table 2 and Supplementary Fig. 1).

**Table 2:** Comparison of bladder intercontraction intervals between PBS and dose-dependently SVF-injected groups

Day	Control	SVF 5 μl	SVF 10 μl	SVF 20 μl	*p*-value
3	51.2	112.0	158.2	184.0	0.011
7	72.0	137.4	175.0	266.4	0.008
14	60.2	144.8	185.0	296.8	0.007

Data are presented as mean and the unit of data is second. SVF, PBS

##### Histology

**Fig. 3:**
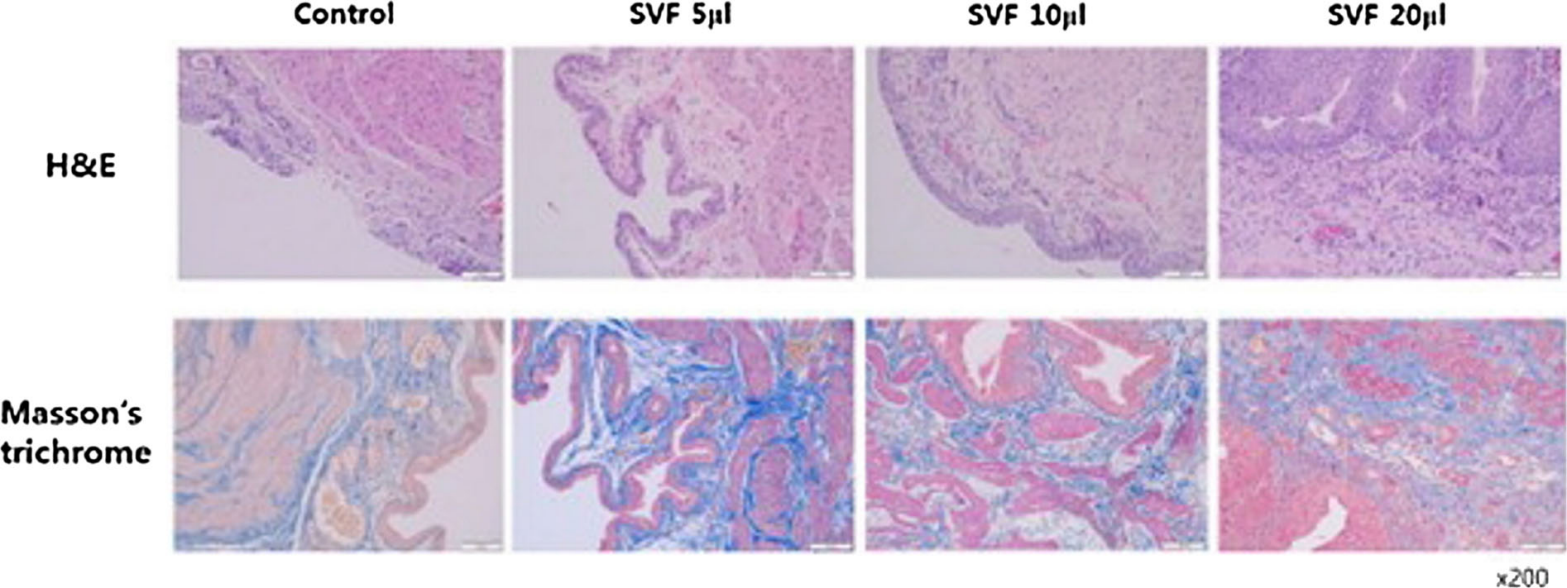
Histological analysis of the SVF or PBS-injected IC bladder. **A** On H&E staining, the bladder section of the control group showed intermittent urothelial denudation, increased inflammatory cell infiltration, and loose connective tissues, while the SVF group had regenerated bladder urothelium and intact lamina propria layers. **B** In Masson’s trichrome staining, fibrotic changes of bladder tissue were observed in all groups, but the density of fibrosis was dose-dependently decreased in the SVF group (blue color) (magnification 200 ×)

**Fig. 4:**
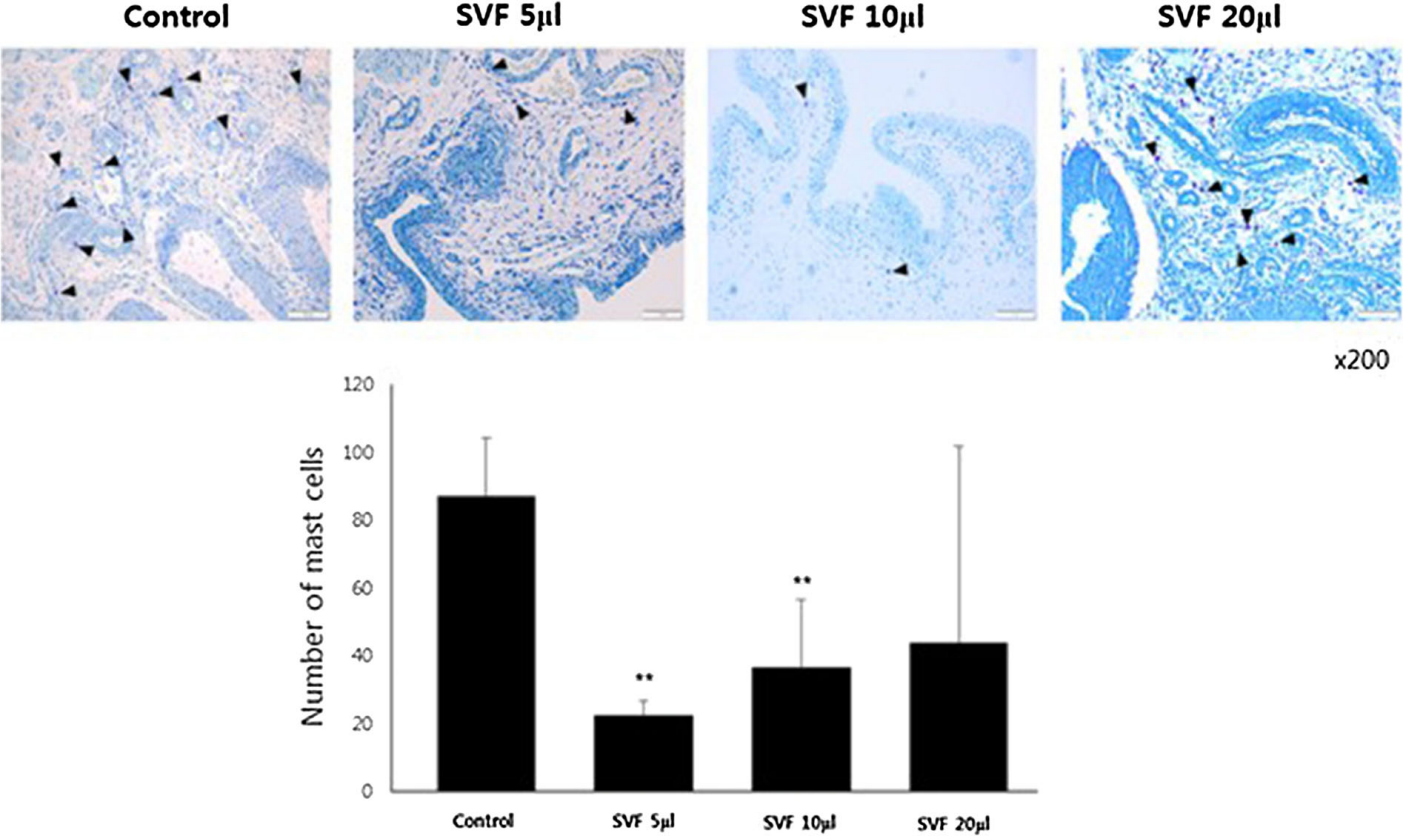
Toluidine blue staining for the quantified analysis of mast cell infiltration. Although significantly decreased numbers of mast cells were observed in the SVF 5 μl and 10 μl groups compared to those in the control group, the mean number of mast cells was the highest in the SVF 20 μl group among all SVF groups. ** above the bar indicates significant differences at *p* < 0.05. Black arrow: mast staining positive cells. Yellow arrow: IHC staining positive cells

H&E staining revealed that intermittent denudation of the urothelium increased inflammatory cell infiltration and loosened connective tissues in the control group. In contrast, the SVF-injected group showed regenerated regular urothelium, intact basement membranes, and lamina propria layers (Fig. 3A). No definite mass-shaped structure was observed in H&E staining. Masson’s trichrome staining revealed fibrotic changes in the bladder in all groups. However, the density of fibrosis (blue) decreased in a dose-dependent manner in the SVF group (Fig. 3B). Bladder sections stained with toluidine blue showed a significantly increased number of mast cells in the lamina propria layer in the control group. Although the number of mast cells in all SVF groups was significantly decreased, the mean number of mast cells was the highest in the SVF 20 μl group among all SVF groups (control, 93.2; SVF 5 μl, 20.9; SVF 10 μl, 35.4; SVF 20 μl, 43.5, p = 0.022) (Fig. 4).

**Fig. 5:**
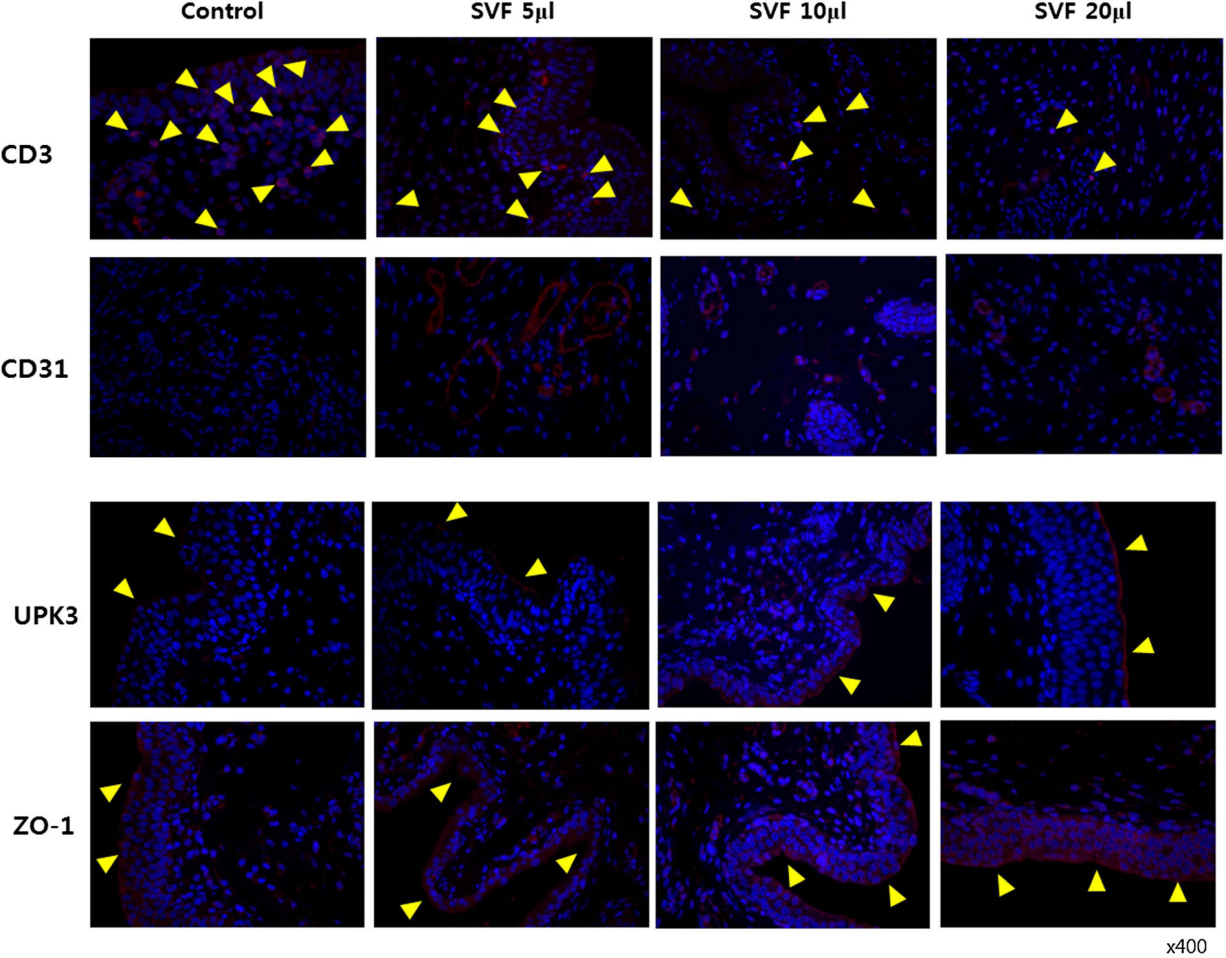
Immunohistochemical staining for the inhibition of inflammatory reactions and bladder tissue regeneration. In the SVF groups, CD3 positive T-cells were significantly reduced dose-dependently compared to those in the control group. CD31, UPK3, and ZO-1 positive areas were more densely observed in all SVF groups in a dose-dependent manner, while they were weakly expressed in the control group. Black arrow: mast staining positive cells. Yellow arrow: IHC staining positive cells

Suppression of the inflammatory reaction (CD3) and regeneration of bladder tissue (CD31, UPK3, and ZO-1) were verified by IHC analysis. CD3-positive T-cells were frequently observed in the control group, whereas they were less frequently observed in the SVF group in a dose-dependent manner, and they were rarely observed in the SVF 20 μl group. CD31-positive cells were rarely observed in the control group, but were strongly enhanced in all SVF groups, regardless of the dose. In a dose-dependent manner, UPK3 and ZO-1 positive areas were also observed to be stronger in all SVF groups, while they were weakly expressed in the control group (Fig. 5).

##### Gene expression

**Fig. 6:**
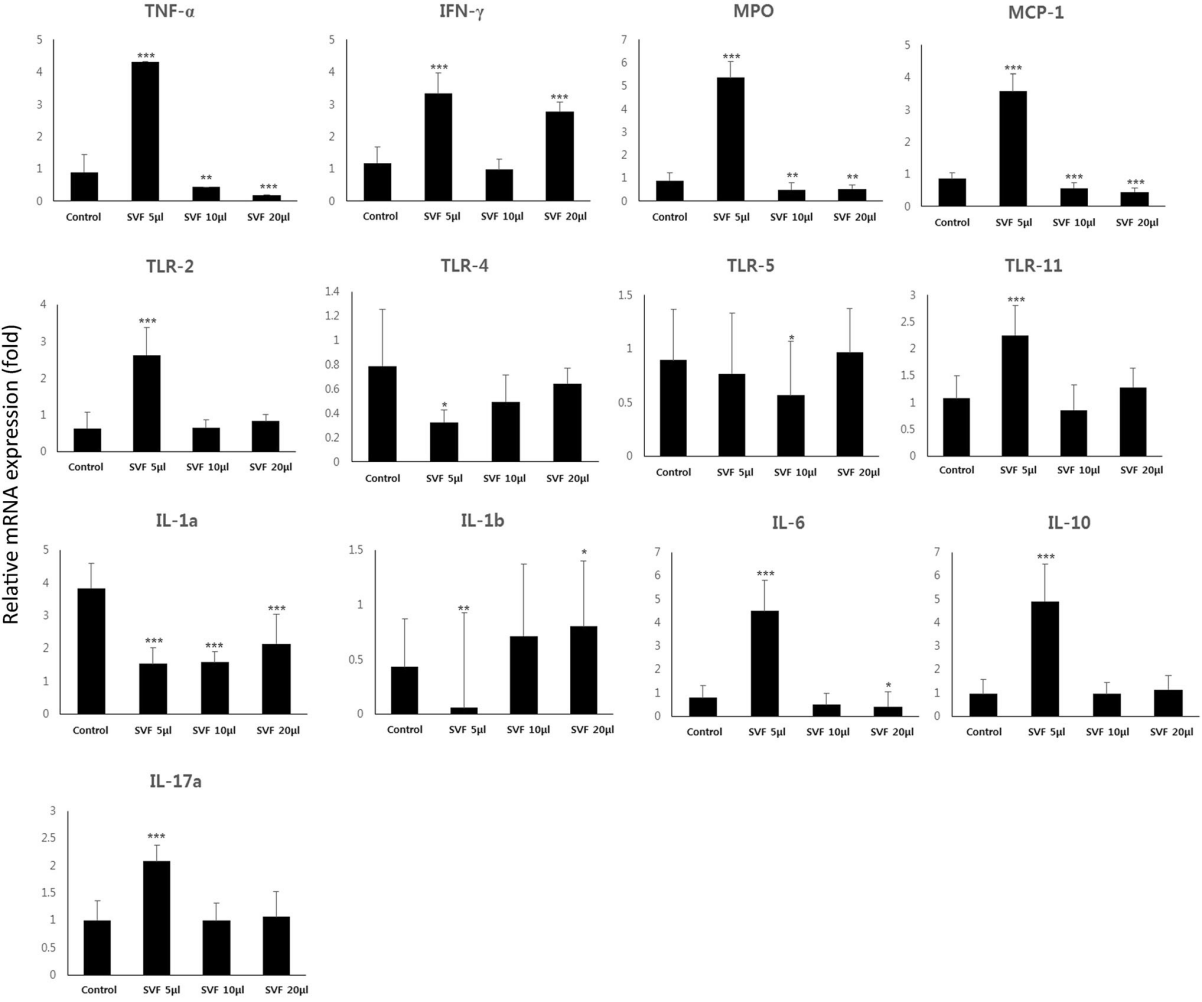
Inflammation-related gene expression analysis using real-time PCR. Expression of TNF-α, MPO, MCP-1, TLR-2, TLR-11, IL-6, IL-10, and IL-17a was significantly highest in the SVF 5 μl group compared to that in the other groups, while it was similar between the other SVF (10 μl and 20 μl) groups and the control group. The expression of IL-1a was significantly lower in all SVF groups compared to that in the control group. ** and *** above the bar indicates significant differences at *p* < 0.01. ** above the bar indicates significant differences at *p* < 0.05. Vertical line : Relative mRNA expression (fold)

To evaluate inflammatory reactions in the bladder after SVF injection, the expression of pro-inflammatory genes was analyzed using real-time PCR. Most of the pro-inflammatory genes, such as TNF-α, MPO, MCP-1, TLR-2, TLR-11, IL-6, IL-10, and IL-17a were significantly highly expressed in the SVF 5 μl group compared to the other groups, while they were similarly expressed in the SVF 10 μl and 20 μl groups compared to the control group. The expression of only IL-1a was significantly lower in all SVF groups than in the control group (Fig. 6).

#### *In vivo* safety

##### Cell tracking

To evaluate the presence or migration of injected adipocyte stem cells from the SVF, we performed 1) qPCR analysis using a human Alu primer and 2) IHC staining with the HuNu antibody. We analyzed human genomic DNA in the bladder, muscle, liver, heart, lung, spleen, kidney, blood, brain, ear, tail, ovary, tooth, and bone marrow of the mice. HuNu was detected in the bladder, muscle, liver, heart, lung, spleen, and kidney tissues. Fresh tissue samples were collected on days 3, 7, 14, and 28 after SVF injection. Human genomic DNA was not detected in any of the collected tissue samples (Supplementary Fig. 2). In addition, no tissue showed positive HuNu staining (Supplementary Fig. 3).

##### Histology

H&E staining was performed to verify the tumorigenicity of the injected SVF and structural changes in the tissue samples (bladder, liver, heart, lung, spleen, and kidney). On screening the H&E-stained tissues, no blood clots or tumor formation was observed (Supplementary Fig. 4). While the control group did not show any inflammatory changes, and lymphocytes or neutrophils were not observed during the entire study period, the IC group showed minimal to mild chronic inflammation with lymphocytes on day 3. The SVF group showed focal acute inflammation with neutrophils on day three. However, these inflammatory changes were not observed after day 14 in either the IC or SVF groups (Supplementary Fig. 4).

## Discussion

IC is a chronic and idiopathic disease usually associated with pelvic pain and bladder storage symptoms such as frequent and urgent voiding [1–3, 7, 15]. Although there are several available treatment modalities, such as behavioral modification, oral medications, intravesical instillation, and hydrodistension, in current clinical practice, most of these treatments offer only temporary symptom relief and do not cure the disease [1, 5]. The etiology and pathophysiology of IC are complicated and not well known, but many studies have suggested that inflammatory changes in the bladder may cause IC [16–18]. Increased infiltration of mast cells is a well-known pathophysiology of IC, and allergic reactions can activate mast cells [19]. Thus, cytokines secreted due to allergic reactions, such as tumor necrosis factor and vasoactive intestinal peptides, are considered to play important roles in IC pathophysiology [20]. Autoimmune reactivity is another possible pathophysiology of IC since autoantibodies against nuclear and bladder urothelium antigens have been found in patients with IC [21]. These inflammatory changes in the bladder mucosa can disrupt the barrier function, and consequently, invasion of urinary constituents into the lamina propria and detrusor muscle layers can occur. Finally, suprapubic or pelvic pain is induced by afferent nerve sensitization [15].

Since the main pathophysiology of IC has been considered to be inflammatory damage of the bladder urothelium, most treatment strategies focus on restoration of the impaired epithelial barrier. Pentosan polysulfate, an approved oral medication for IC, has a pharmacological effect on restoring the damaged glycosaminoglycan layer of the bladder mucosa [22]. Intravesical therapy using hyaluronic acid, which is approved as a standard therapy for IC in several countries, also targets the restoration of the glycosaminoglycan layer [23]. However, the effectiveness of current treatments is limited, and they do not cure the disease [24].

To overcome these limitations, stem cell therapy using MSCs has recently been proposed, and several preclinical studies have shown promising results. In 2014, Chen et al. administered melatonin and ADSCs to a cyclophosphamide-induced IC rat model and demonstrated their anti-inflammatory effects in the IC rat model [4]. Song et al. reported the therapeutic effects of human umbilical cord blood-derived MSCs in a hydrochloric acid-induced IC rat model via activation of the WNT signaling pathway [5]. Xiao et al. analyzed the effects of bone marrow-derived MSCs in an IC rat model via the TGF-β/MAPK signaling pathway [25]. Xie et al. confirmed that umbilical cord blood reduced inflammation, inhibited apoptosis, and promoted proliferation in a cyclophosphamide-induced IC rat model by activating the AKT/mTOR signaling pathway [6]. In a uroplakin-induced IC rat model, Chung et al. reported that human tissue-derived MSCs, regardless of the cell source, could regenerate damaged bladder tissue, inhibit inflammatory cell accumulation, and promote functional recovery of the bladder [7].

Stem cells are known to exist in various organs and tissues and have self-renewal ability and the potential to differentiate into progenitor cells to regenerate damaged cells or tissues [26]. Among these various source-derived MSCs, ADSCs are known to be one of the easiest stem cells to isolate from the human body and are relatively abundant in quantity as well. Up to approximately 500 times more stem cells per gram of ADSCs can be procured than bone marrow-derived stem cells [27]. Therefore, ADSCs have been used for the regeneration of various organs and tissues in many preclinical studies [28]. Although ADSCs have shown promising potential for organ or tissue regeneration, there are still several limitations to their clinical application, such as the culture process. Because of these limitations, the SVF has recently been introduced and widely investigated [9].

The SVF is a biological product isolated from the enzymatic or non-enzymatic (e.g., mechanical agitation) digestion of adipose tissue, which is split into fat and stromal/vascular fractions. The bottom layer is referred to as the SVF. The Ustem kit is a commercial product designed to simplify the separation process. The SVF contain stem cells and cytokines, which are associated with regenerative, antinociceptive, immunomodulatory, and anti-inflammatory effects [10]. Thus, the SVF has several advantages over pure ADSCs, as the SVF has a distinct and heterogeneous cellular composition compared to that of MSCs, The SVF has demonstrated superior therapeutic results in several preclinical studies [29–32]. In addition, the SVF is more easily acquired than ADSCs without the process of cell separation or culture conditions [9].

Although ADSCs have traditionally been derived from subcutaneous fat, which mainly contains white adipose tissue, the value of brown adipose tissue has recently been enhanced [11, 33, 34]. Brown adipose tissue is known to redundantly exist in perirenal adipose tissue, especially around the adrenal gland [11]. The main role of brown adipose tissue, which is mainly distributed in the perirenal adipose tissue, is to maintain the body temperature [11]. In addition, activated brown adipose cells secrete substances via the endocrine pathway and affect other metabolic tissues to regulate energy metabolism and inflammation [8, 35]. Brown adipose tissue is also associated with circulating exosomal miRNAs. Brown fat tissue secretes exosomal miRNAs that inhibit transcription [11].

Kidney surgery, including nephrectomy, is performed worldwide, and perirenal fat tissue is regarded as medical waste and is usually discarded. Therefore, perirenal adipose tissue-derived ADSCs or SVF can be collected in a cost-effective manner without ethical issues. However, only a few studies have used perirenal adipose tissue-derived SVF or ADSCs. Therefore, this study aimed to isolate the SVF from perirenal adipose tissue, which was procured from a healthy kidney donor, and analyzed the efficacy and safety of the SVF in an IC rat model.

Therefore, it is necessary to establish an appropriate animal model to investigate new therapeutic modalities. As the exact pathophysiology of IC is not clearly understood, approximately 20 animal models with characteristics similar to those of the IC phenotype have been generated [3]. However, Song et al. recently suggested that injecting uroplakin generated the most effective IC animal model [3], and Kim et al. suggested that injection of uroplakin3A could generate an IC animal model with a painful bladder [1]. Because bladder pain is one of the main phenotypes of IC, it is necessary to evaluate pain scales to verify SVF. Thus, the IC rat model was generated by subcutaneous injection of uroplakin3A.

Most studies related to stem cell therapy using MSCs in animal models of IC have shown functional improvement of the bladder with anti-inflammatory and anti-fibrotic effects [1, 5, 7]. In this study, functional improvement of the bladder, such as bladder pain and detrusor overactivity, was significantly seen after SVF injection, but anti-inflammatory and anti-fibrotic changes were not dramatically improved. The main regenerative factor of the SVF is adult stem cells, which have a function similar to that of MSCs. The mechanism of stem cells in the repair of damaged tissues is multifactorial. One possible mechanism is the formation of target cells by direct stem cell engraftment and differentiation in a target organ. Another mechanism is the paracrine effect, meaning that stem cells stimulate complex cellular signaling systems, which can induce angiogenesis, promote cell survival, and prevent cellular apoptosis in damaged tissues [10]. These mechanisms can explain the results of both MSCs and the SVF for functional and structural improvement of the bladder in IC animal models. Although the SVF is known to contain T-reg cells that may be helpful in immunomodulation, there are several other types of cells and cytokines in the SVF [10]. This might have negative effects on the control of inflammation in an IC animal model, as the SVF was derived from human adipose tissue in this study. Therefore, the SVF has been suggested for use in autologous treatments [9].

Nevertheless, in this study, a non-autologous adipose tissue-derived SVF was administered in an animal model, and the efficacy of the SVF in terms of functional improvement was observed. In addition, a sufficient amount of the autologous SVF may not be procured from subcutaneous fat in some patients, such as those with low body weight. For these reasons and the potential benefit of perirenal adipose tissue-derived SVF, investigation of the use of an allogeneic adipose tissue-derived SVF is needed. Similar to organ transplantation, if the immunomodulatory effect can be acquired from an allogenic SVF using a specific preparation during SVF isolation or by administering local or systemic immunosuppressants, a widely available source for organ and tissue regeneration can be established.

Since the first clinical application of the SVF for cosmetic breast augmentation in 2007 by Rigotti et al. [36], many preclinical studies and clinical trials are underway [9]. Recently, Lander et al. performed a clinical evaluation of an autologous SVF in IC. In their study, 109 IC patients were enrolled, and the SVF was administered both intravenously and regionally to the pelvic floor targets. Of these, 78 patients (71.6%) reported improvement in their IC-related symptoms [10]. Although this study showed early positive clinical results of the SVF for IC, the patients were only assessed with symptom questionnaires, and the mechanism of the SVF for IC has not been clearly discussed. Therefore, preclinical evaluation of the SVF in an IC rat model was performed in this study, not only to verify the efficacy and safety of the SVF for IC treatment but also to evaluate the histological changes of the bladder and the expression of inflammatory markers. This study demonstrated that the SVF has promising efficacy for the functional improvement of the IC bladder, but the inhibitory effect on inflammation is not certain. Thus, it remains unclear if the SVF has long-term therapeutic effects or can even cure IC.

Therefore, it is necessary to evaluate the long-term effects of the SVF in IC animal models or in IC patients, and further well-designed clinical trials, such as randomized controlled trials, should be performed to verify the efficacy of the SVF for IC in the human body. Moreover, further research should be performed to enhance the action of the SVF* in vivo*, and combination therapy using the SVF and other medications or treatment modalities should be investigated.

This study has several limitations. The first is the use of a non-autologous adipose-tissue-derived SVF. Unlike MSCs, the SVF is known not to have an immunomodulatory effect; therefore, most studies have used an autologous fat-derived SVF in their research. However, this study showed that a human adipose tissue-derived SVF could induce functional improvement of the bladder even in a non-immunodeficient IC rat model. Based on these results, the possible use of a non-autologous fat-derived SVF is expected in the future. Although the efficacy and safety of a perirenal adipose tissue-derived SVF in an IC rat model were verified in this study, a comparison with pure ADSCs or a subcutaneous adipose tissue-derived SVF was not performed. A comparative study with previously verified regenerative cell sources can be helpful in observing the superiority or inferiority of the perirenal fat-derived SVF, but the verification of the perirenal fat-derived SVF itself also has value with the distinctive advantages of visceral fat and the sufficiency of discarded perirenal adipose tissues. Immune-deficient mice were used to evaluate the safety of the perirenal adipose tissue-derived SVF. In this animal setting, the exact mechanism of the immune reaction using the human adipose tissue-derived SVF could not be analyzed. However, since the mechanism of a xenogenic tissue-derived SVF is still not well known, nude mice were selected for long-term safety analysis of the SVF in terms of tumorigenicity and thrombus formation.

## Conclusion

IC is a chronic intractable disease for which there are no effective and curative treatments. Recent studies using stem cells for IC have shown promising results in cell therapy, and the introduction of the SVF has accelerated the clinical application of stem cell therapy. This study demonstrated that the SVF improved bladder pain and overactivity in an IC rat model and that both subcutaneous fat and perirenal adipose tissue can be a good source of an SVF. Considering the advantages of perirenal adipose tissue and the limitations of subcutaneous fat tissue in skinny patients, further use of perirenal adipose tissue in regenerative medicine is expected. However, for the effective and safe clinical applications of ADSCs and the SVF, a better understanding of ADSC and SVF biology should be achieved, and further well-designed clinical trials should be performed in the near future.

## Supplementary Information

Below is the link to the electronic supplementary material.

Supplementary file1 (JPG 72 KB)

Supplementary file2 (JPG 813 KB)

Supplementary file3 (JPG 764 KB)

Supplementary file4 (JPG 787 KB)

Supplementary file5 (JPG 1893 KB)

Supplementary file6 (JPG 2365 KB)
