# Supplementary figures and images for: The Efficacy and Safety of a Human Perirenal Adipose Tissue-Derived Stromal Vascular Fraction in an Interstitial Cystitis Rat Model
Source: Tissue Eng Regen Med. 2023 Jan 4;20(2):225–37. doi: 10.1007/s13770-022-00505-w (PMC10070579; doi:10.1007/s13770-022-00505-w)

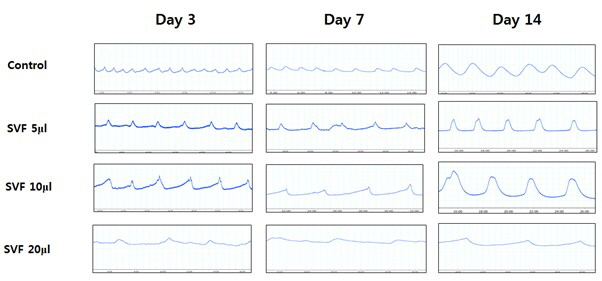

Supplement: Supplementary file 1 — Supplementary file1 (JPG 72 KB) [file 13770_2022_505_MOESM1_ESM.jpg]

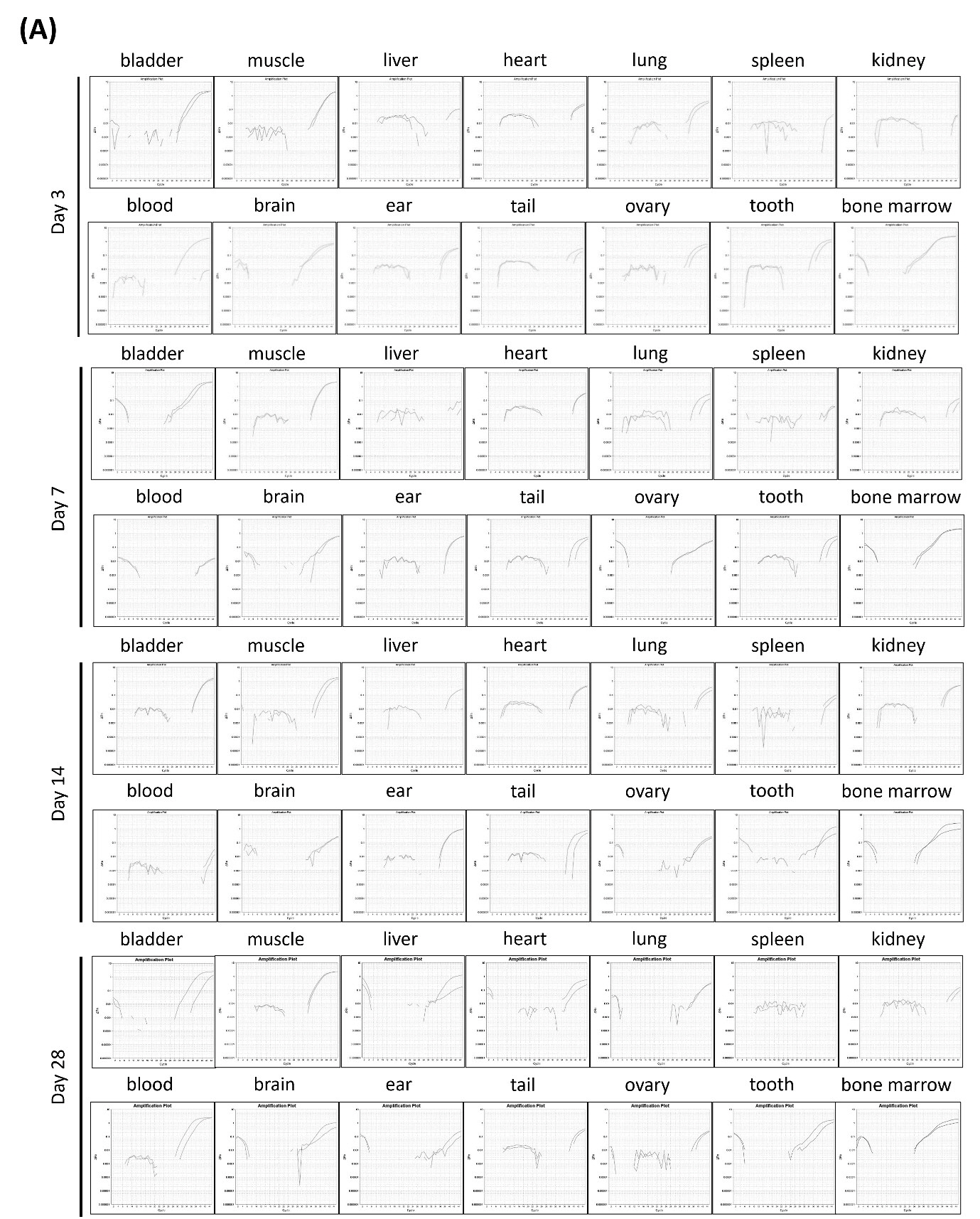

Supplement: Supplementary file 2 — Supplementary file2 (JPG 813 KB) [file 13770_2022_505_MOESM2_ESM.jpg]

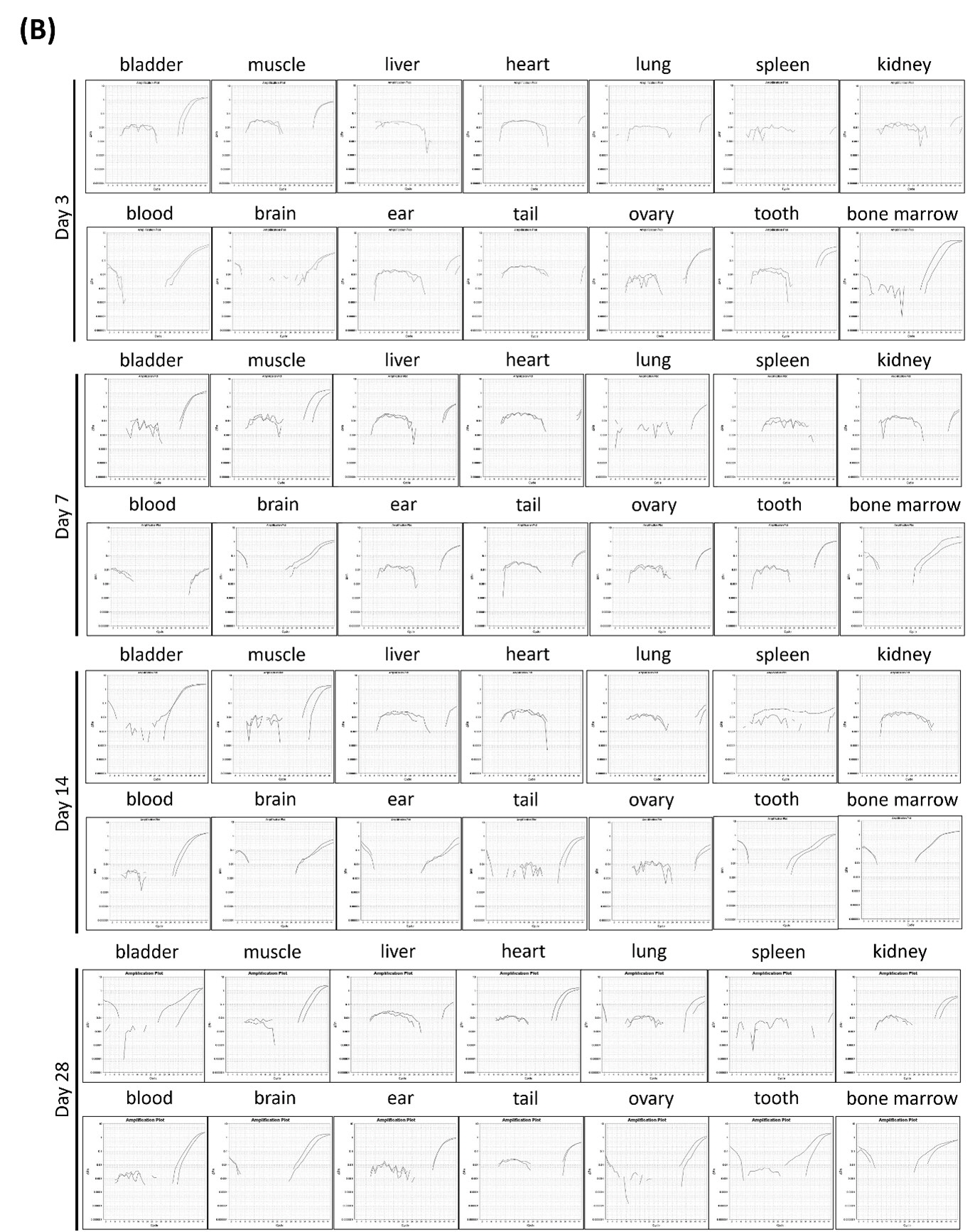

Supplement: Supplementary file 3 — Supplementary file3 (JPG 764 KB) [file 13770_2022_505_MOESM3_ESM.jpg]

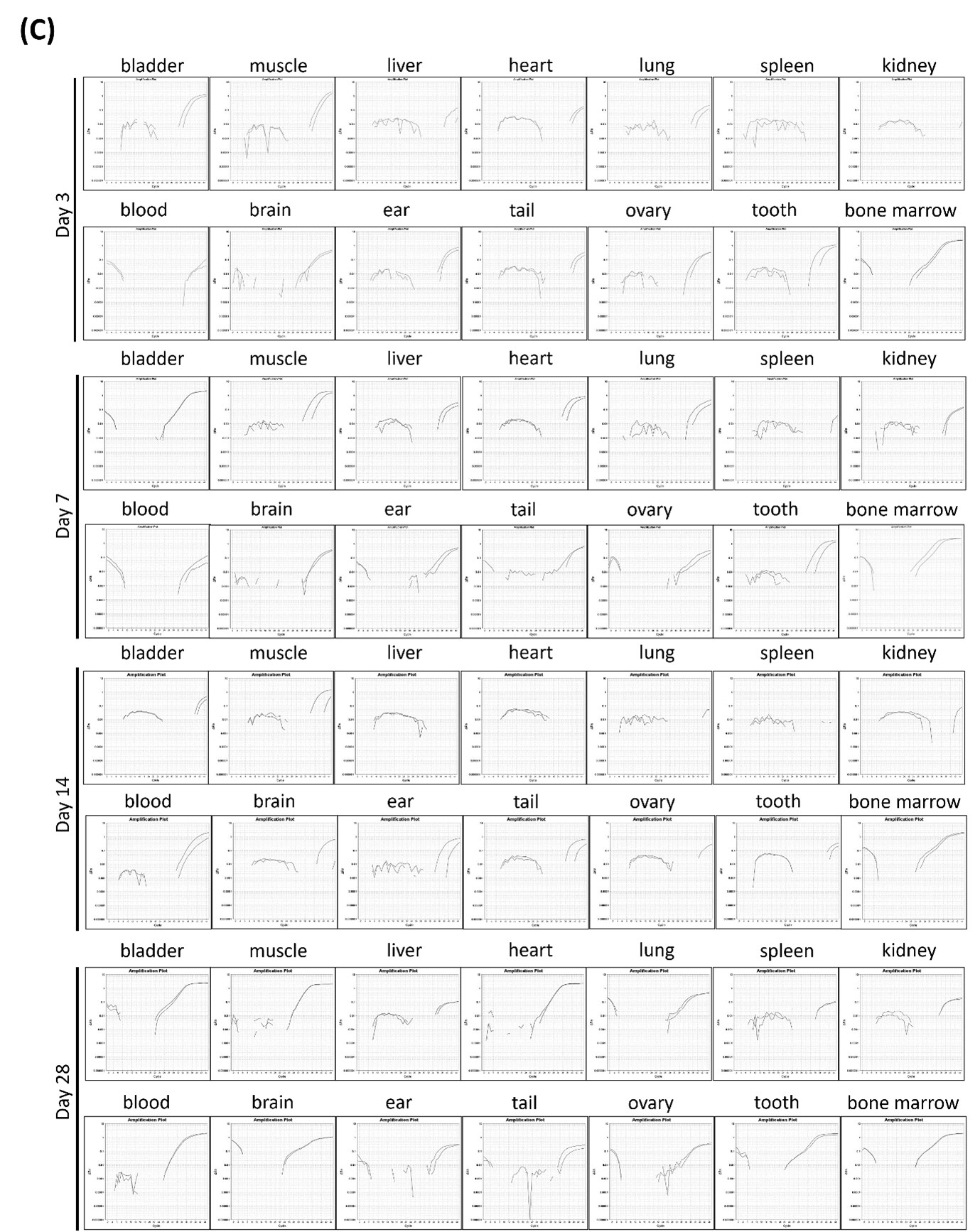

Supplement: Supplementary file 4 — Supplementary file4 (JPG 787 KB) [file 13770_2022_505_MOESM4_ESM.jpg]

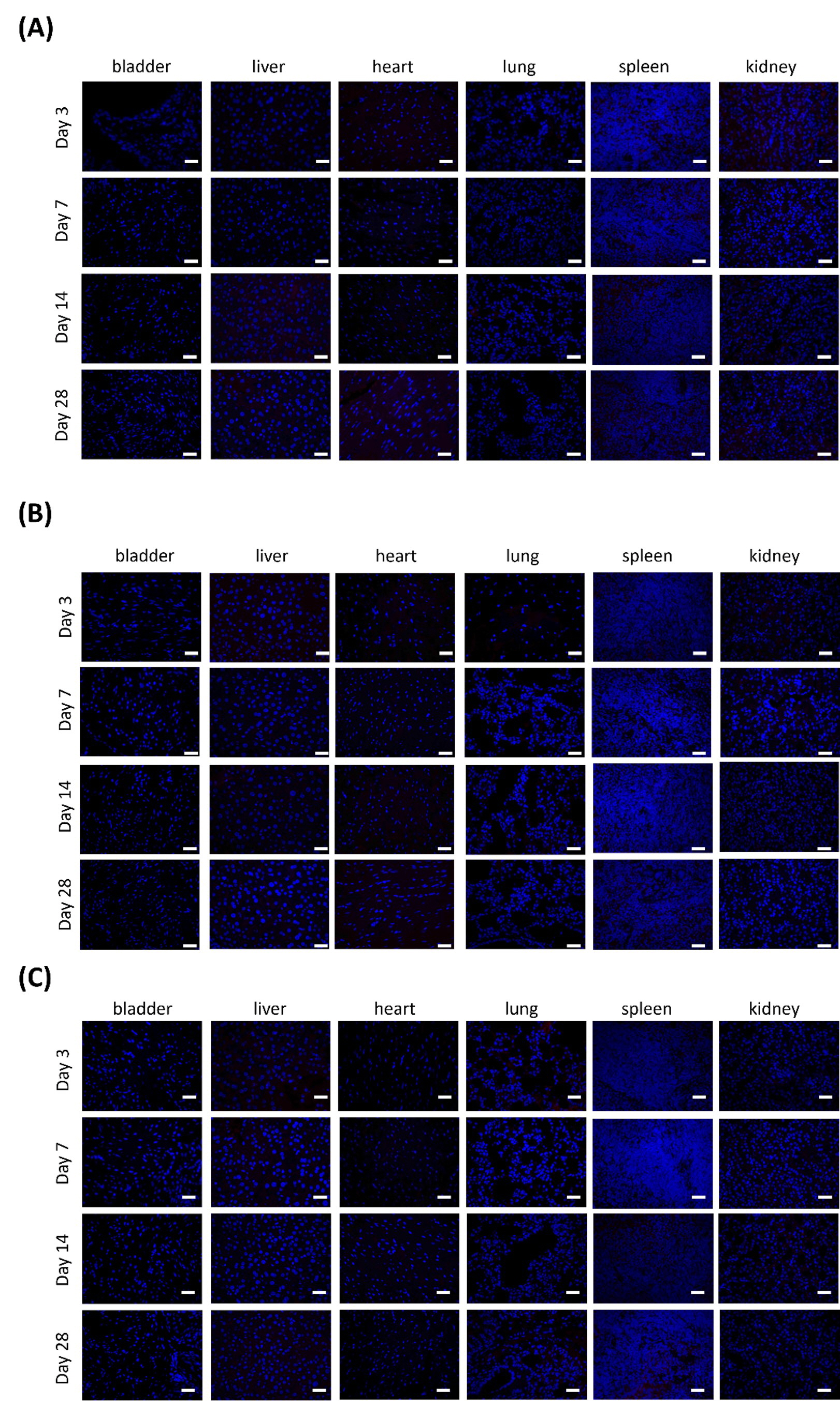

Supplement: Supplementary file 5 — Supplementary file5 (JPG 1893 KB) [file 13770_2022_505_MOESM5_ESM.jpg]

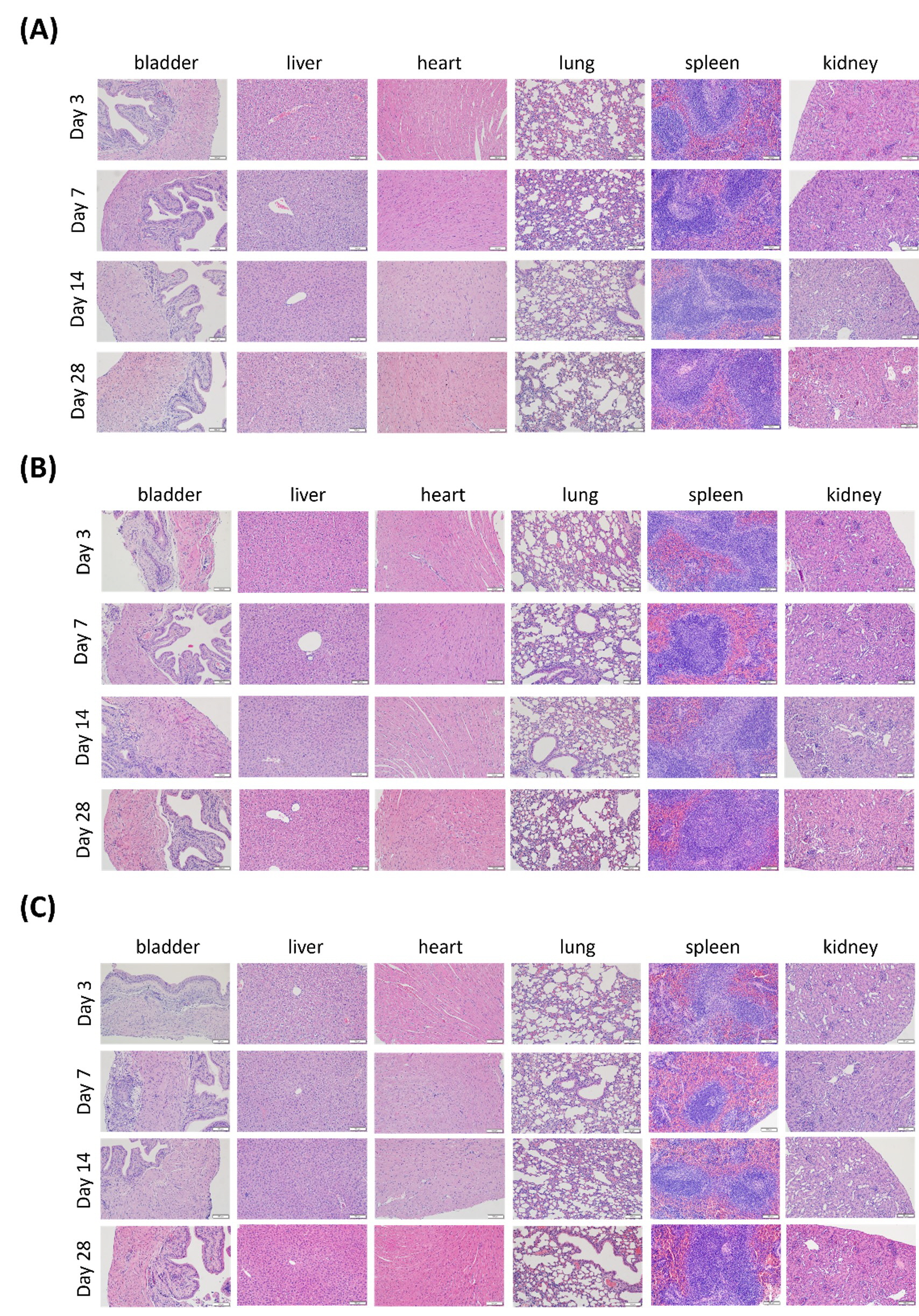

Supplement: Supplementary file 6 — Supplementary file6 (JPG 2365 KB) [file 13770_2022_505_MOESM6_ESM.jpg]
